# The molecular pathogenesis of achalasia: a paired lower esophageal sphincter muscle and serum 4D label-free proteomic study

**DOI:** 10.1093/gastro/goad031

**Published:** 2023-06-12

**Authors:** Songfeng Chen, Xiangbin Xing, Xun Hou, Qianjun Zhuang, Niandi Tan, Yi Cui, Jinhui Wang, Mengyu Zhang, Shixian Hu, Yinglian Xiao

**Affiliations:** Department of Gastroenterology, The First Affiliated Hospital of Sun Yat-sen University, Guangzhou, Guangdong, P. R. China; Department of Gastroenterology, The First Affiliated Hospital of Sun Yat-sen University, Guangzhou, Guangdong, P. R. China; Gastrointestinal Surgery Center, The First Affiliated Hospital of Sun Yat-sen University, Guangzhou, Guangdong, P. R. China; Department of Gastroenterology, The First Affiliated Hospital of Sun Yat-sen University, Guangzhou, Guangdong, P. R. China; Department of Gastroenterology, The First Affiliated Hospital of Sun Yat-sen University, Guangzhou, Guangdong, P. R. China; Department of Gastroenterology, The First Affiliated Hospital of Sun Yat-sen University, Guangzhou, Guangdong, P. R. China; Department of Gastroenterology, The First Affiliated Hospital of Sun Yat-sen University, Guangzhou, Guangdong, P. R. China; Department of Gastroenterology, The First Affiliated Hospital of Sun Yat-sen University, Guangzhou, Guangdong, P. R. China; Institute of Precision Medicine, The First Affiliated Hospital of Sun Yat-sen University, Guangzhou, Guangdong, P. R. China; Department of Gastroenterology, The First Affiliated Hospital of Sun Yat-sen University, Guangzhou, Guangdong, P. R. China

**Keywords:** achalasia, proteomics, serum, lower esophageal sphincter

## Abstract

**Background:**

Achalasia is a primary esophageal motility disorder with potential molecular pathogenesis remaining uncertain. This study aimed to identify the differentially expressed proteins and potential pathways among achalasia subtypes and controls to further reveal the molecular pathogenesis of achalasia.

**Methods:**

Paired lower esophageal sphincter (LES) muscle and serum samples from 24 achalasia patients were collected. We also collected 10 normal serum samples from healthy controls and 10 normal LES muscle samples from esophageal cancer patients. The 4D label-free proteomic analysis was performed to identify the potential proteins and pathways involved in achalasia.

**Results:**

Analysis of Similarities showed distinct proteomic patterns of serum and muscle samples between achalasia patients and controls (both *P *<* *0.05). Functional enrichment analysis suggested that these differentially expressed proteins were immunity-, infection-, inflammation-, and neurodegeneration-associated. The mfuzz analysis in LES specimens showed that proteins involved in the extracellular matrix–receptor interaction increased sequentially between the control group, type III, type II, and type I achalasia. Only 26 proteins altered in the same directions in serum and muscle samples.

**Conclusions:**

This first 4D label-free proteomic study of achalasia indicated that there were specific protein alterations in both the serum and muscle of achalasia, involving immunity, inflammation, infection, and neurodegeneration pathways. Distinct protein clusters between types I, II, and III revealed the potential molecular pathways associated with different disease stages. Analysis of proteins changed in both muscle and serum samples highlighted the importance of further studies on LES muscle and revealed potential autoantibodies.

## Introduction

Achalasia is a primary esophageal motility disorder characterized by the aberrant peristalsis of the esophageal body and insufficient relaxation of the lower esophageal sphincter (LES) [1, 2]. Epidemiological studies showed that the global incidence and prevalence of achalasia are 0.03–1.63 and 1.8–12.6 per 100,000 persons per year, respectively [3]. According to Chicago classification version 4.0, achalasia can be further divided into three different subtypes [4]. Although all subtypes present integrated relaxation pressure elevated under manometry examination, the esophageal peristalsis pattern (type I: 100% failed contractions; type II: ≥20% panesophageal pressurization; type III: ≥20% spasm contractions) [4] and the clinical prognosis (type II is the best while type III is the worst) [5] of different subtypes of achalasia differ greatly. Achalasia not only causes dysphagia and other symptoms that impair patients’ quality of life; it also causes chronic esophageal inflammation by food retention and eventually increases the risk of esophageal cancer [6].

The treatments for achalasia aim to relieve the obstruction of LES. This goal can be accomplished by a variety of methods such as calcium channel blockers, nitrates, laparoscopic myotomy, per-oral endoscopic myotomy (POEM), pneumatic dilation, or Botox injection [3]. However, all of the current treatments impair the normal anti-reflux barrier, which may lead to gastroesophageal reflux disease (∼35.3% of achalasia patients presented esophagitis after treatment) [7, 8]. Furthermore, these treatments are also palliative and often require repeated treatments (the mid-term recurrence rate of POEM was ∼18.0%) [9], which incurs huge medical expenditure. It was estimated that achalasia incurs >$408 million direct medical costs in the USA annually [10]. Therefore, there is an urgent need to further investigate the pathogenesis of achalasia to find new therapeutic targets.

LES has been reported to be involved in achalasia [11, 12]. Previous histopathology studies have demonstrated that inhibitory neurons (mainly neuronal nitric oxide synthase [nNOS] positive cells) in LES muscle in achalasia patients were degenerative [12–14]. A genetic deficiency animal study also found that the reduction of nNOS can cause LES dysfunction and esophageal body peristalsis disorder [15]. Although the degeneration of inhibitory neurons in achalasia patients has become a consensus, the causes are still unknown. One possible hypothesis is that a genetically susceptible population potentially have higher risk of infection with herpes virus, triggering autoimmune response to inhibitory neurons in LES [11, 13]. This hypothesis is based on the following research findings: first, multiple studies have found a higher detection rate of virus DNA in the serum or LES muscle of achalasia patients [16, 17]; second, patients with achalasia exhibited increased levels of immune-related serological cytokines and chemokines [18]; histopathological studies also found that in patients with achalasia, mast cell infiltration and eosinophil cell infiltration in the LES muscle were increased [19, 20]; lastly, by using indirect immunofluorescence and immunoblotting assay, some studies have found potential antineuronal antibodies in achalasia patients [21, 22]. However, these pieces of research were all limited to certain aspects of the disease and lack systematic screening.

In order to gain a fuller understanding of achalasia, some studies have been conducted to identify achalasia-associated molecular traits from both genomic and transcriptomic levels. Whole-exome sequencing studies and genetic association studies have found susceptibility genes (rs1705003, rs1126511, and rs28688207) for achalasia [23, 24]. Transcriptomic studies in LES muscle found that molecules involved in immunity, skeletal, and muscular system development and nervous system development macro-processes were over-represented in achalasia patients [25, 26]. However, there is still a lack of protein evidence that is more pronounced in this disease due to its direct functionality. Moreover, none of the current pathogenesis studies can explain the heterogeneity among different subtypes of achalasia.

By using a 4D label-free quantification tool, a high-throughput proteomic measurement, we comprehensively characterized the difference in tissue and serum proteins between achalasia patients and controls. The aim of this study was to identify the differentially expressed proteins and potential pathways across achalasia subtypes and controls to further reveal the molecular pathogenesis of achalasia.

## Methods

### Study population

**Figure 1.**
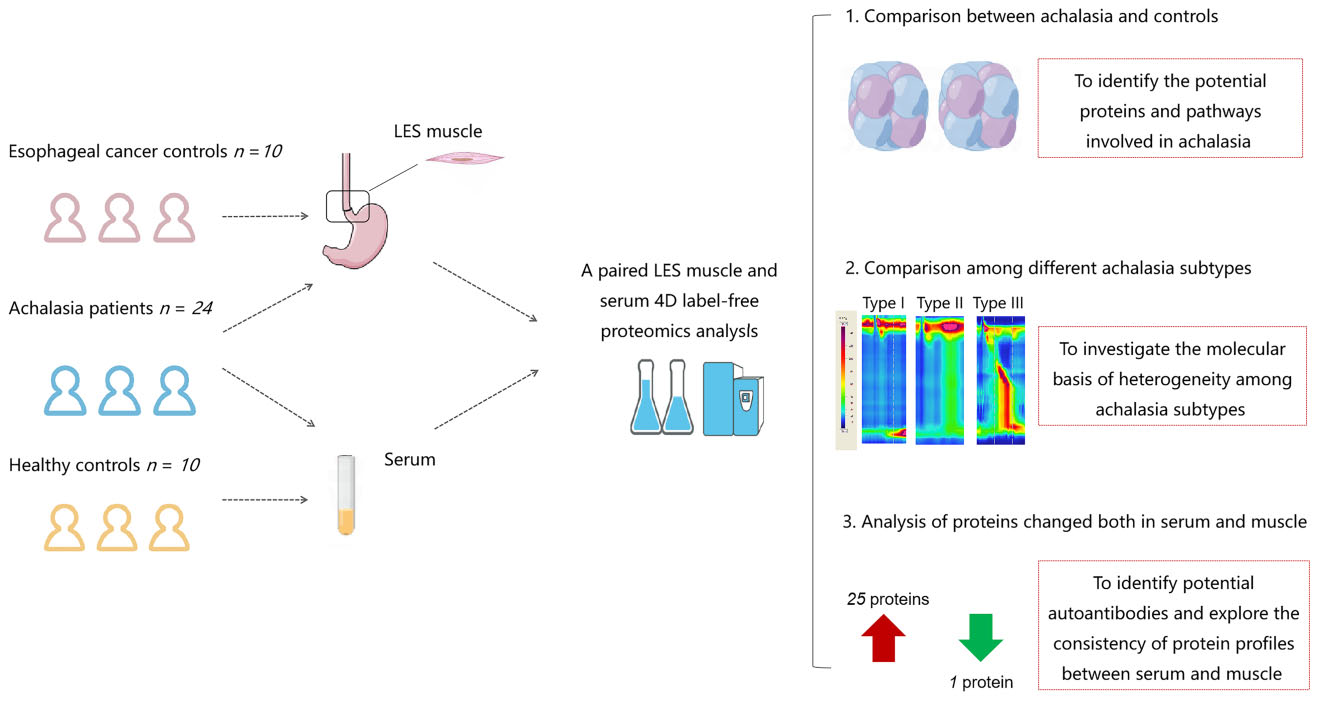
Flow diagram showing the study design. LES, lower esophageal sphincter.

Patients who were diagnosed with achalasia by using high-resolution manometry and received POEM in the First Affiliated Hospital of Sun Yat-sen University (Guangzhou, China) from September 2021 to March 2022 were included. Patients who met any of the following criteria were excluded: (i) patients with other known organic disease of the digestive tract; (ii) patients who had received previous upper gastrointestinal tract invasive treatments; (iii) patients with history of digestive tumor; (iv) patients with rheumatic or immune system disease; (v) patients with current infection; (vi) patients who had received antibiotic or proton-pump inhibitor 8 weeks prior to the POEM procedure. Achalasia patients were divided into three different subtypes according their manometry manifestation prior to the POEM procedure following the criteria of Chicago classification version 4.0 [4]. We also included 10 healthy volunteers who had had no upper digestive symptoms 6 months prior and had received no medication 8 weeks prior to our study to obtain normal serum controls. To obtain normal LES muscle controls, 10 esophageal cancer patients whose tumors were ≥5 cm away from the LES were included. The flow diagram of this study is shown in Figure 1.

This study was approved by the Ethical Review Board of Sun Yat-sen University (IRB no. 2022405) and was conducted in accordance with the Declaration of Helsinki.

### Specimen collection procedure

For achalasia patients, after one night of fasting, a 10-mL peripheral blood sample was collected before the POEM procedure and three esophageal LES muscle samples (0.5 cm each) were collected using biopsy forceps after myotomy during the POEM procedure. For esophageal cancer patients, three normal LES muscle specimens (pathologically confirmed as normal) were collected after the esophagectomy. For healthy volunteers, a 10-mL peripheral blood sample was collected after one night of fasting. Fasting peripheral blood samples were centrifuged (1,200 ×*g*, 10 min, 4°C) and the serum layers were collected and stored in a −80°C refrigerator. LES muscle specimens were frozen in liquid nitrogen immediately after collection and then stored in the −80°C refrigerator until protein extraction.

### Protein extraction and trypsin digestion

For LES muscle specimens, the samples were ground using liquid nitrogen into a cell powder and then transferred to a 5-mL centrifuge tube. After that, lysis buffer (8 M urea, 1% protease inhibitor cocktail) was added to the cell powder, followed by sonication three times on ice. The remaining debris was removed by using centrifugation at 12,000 ×*g* at 4°C for 10 min and the supernatant was collected.

For serum samples, the top 14 high abundance proteins were removed by using a Pierce^TM^ Top 14 Abundant Protein Depletion Spin Columns Kit (Thermo Fisher Scientific, Waltham, USA).

The protein solution was reduced using 5 mM of dithiothreitol for 30 min at 56°C and alkylated with 11 mM of iodoacetamide for 15 min at 37°C in darkness. Then, the protein sample was diluted by adding 100 mM of tetraethyl ammonium bromide to a urea concentration of <2 M. After that, trypsin was added at a 1:50 trypsin-to-protein mass ratio for the first digestion overnight and 1:100 trypsin-to-protein mass ratio for a second 4 h of digestion.

### Tandem mass tag labeling

Trypsin enzymatic peptides were desalted using Strata X C18 (Phenomenex, Los Angeles, USA) and freeze-dried. The peptides were marked according to the tandem mass tag kit instructions (Thermo Fisher Scientific, Waltham, USA).

### Liquid chromatography tandem mass spectrometry (LC–MS/MS) analysis and database searching

The tryptic peptides were dissolved in solvent A (0.1% formic acid, 2% acetonitrile/in water) directly loaded onto a home-made reversed-phase analytical column (25 cm in length, 75/100 μm inside diameter (i.d.)). Peptides were separated with a gradient from 6% to 24% solvent B (0.1% formic acid in acetonitrile) over 70 min, 24% to 35% in 14 min and climbing to 80% in 3 min then holding at 80% for the last 3 min, all at a constant flow rate of 450 nL/min on a nanoElute UHPLC system (Bruker Daltonics, Beijing, China).

The peptides were subjected to capillary source followed by the timsTOF Pro (Bruker Daltonics, Beijing, China) mass spectrometry. The electrospray voltage applied was 1.60 kV. Precursors and fragments were analysed at the time of flight (TOF) detector, with a MS/MS scan range from 100 to 1,700 m/z. The timsTOF Pro was operated in parallel accumulation–serial fragmentation (PASEF) mode. Precursors with charge states 0–5 were selected for fragmentation and 10 PASEF-MS/MS scans were acquired per cycle. The dynamic exclusion was set to 30 s.

The resulting data were processed using the MaxQuant search engine (v.1.6.15.0). Tandem mass spectra were searched against the human SwissProt database (20,422 entries) concatenated with a reverse decoy database. Trypsin/P was specified as the cleavage enzyme allowing up to two missing cleavages. The mass tolerance for the precursor ions was set as 40part per million (p.p.m) in the first search and 40 p.p.m. in the main search, while the mass tolerance for fragment ions was set as 0.04 Da. Carbamidomethyl on cysteine was specified as a fixed modification, and acetylation on protein N-terminal and oxidation on methionine were specified as variable modifications. The false discovery rate was set at <1%.

### Statistical analysis

Principal component analysis (PCA) and Analysis of Similarities (ANOSIM) were used to assess the protein expression pattern difference between patients with achalasia and controls. The mfuzz analysis was used to analyse the trend of protein changes in different achalasia subtypes and the function of these proteins. Unlike hard clustering, which only allows each gene or protein to be assigned to one cluster, the mfuzz analysis is a soft clustering method that allows overlaps between gene or protein clusters and is more noise-robust. Proteins of the same cluster had similar expression trends among groups [26]. Differentially expressed proteins were assessed by using a *t*-test and further filtered based on the following criteria: (i) ratio > 1.5/1 or < 1/1.5; (2) *P *<* *0.05; (3) detection rate of >20% per group. The Gene Ontology (GO) annotation and the Kyoto Encyclopedia of Genes and Genomes (KEGG) annotation were also performed to investigate the potential pathways [27].

For demographic characterization, percentage and chi-square test were used for categorical variables. For continuous variables, if the variables were normally distributed, one-way analysis of variance (ANOVA) analysis was used, otherwise the Kruskal–Wallis test was used. The significant difference was set at *P *<* *0.05.

## Results

A total of 24 achalasia patients, 10 healthy volunteers, and 10 esophageal cancer patients were included in the current study. Among patients with achalasia, 10 were diagnosed with type I, 10 with type II, and 4 with type III by using high-resolution manometry according to Chicago classification version 4.0 [4]. No significant difference was found in the demographic data between achalasia patients, healthy volunteers, and esophageal cancer patients (Table 1).

**Table 1.** Baseline data comparison among achalasia patients and controls

Characteristic	Type I (*n *=* *10)	Type II (*n *=* *10)	Type III (*n *=* *4)	Healthy volunteer (*n *=* *10)	Esophageal cancer patient (*n *=* *10)	*P*-value
Male, *n* (%)	6 (60)	4 (40)	2 (50)	5 (50)	6 (60)	0.892
Age, years, mean ± SD	48.2 ± 14.6	54.3 ± 12.4	44.8 ± 11.8	46.5 ± 16.8	41.8 ± 17.2	0.469
BMI, kg/m^2^, mean ± SD	17.8 ± 1.7	19.6 ± 2.8	18.5 ± 2.3	17.8 ± 1.7	17.7 ± 2.3	0.234
Disease duration, months, median (IQR)	48.0 (11.3–135.0)	36.0 (11.5–120.0)	96.0 (36.0–120.0)	–	–	0.560
Eckardt score, mean ± SD	6.1 ± 2.2	5.7 ± 2.4	6.3 ± 2.5	–	–	0.893
LES basal pressure, mmHg, median (IQR)	23.1 (14.5–43.7)	27.0 (23.3–34.8)	27.1 (25.2–43.1)	–	–	0.437
Integrated relaxation pressure, mmHg, mean ± SD	22.6 ± 3.9	27.0 ± 6.4	24.6 ± 5.8	–	–	0.218

BMI, body mass index; IQR, interquartile range; LES, lower esophageal sphincter; SD, standard deviation.

### Overview of proteins identified

The peptides were distributed in 7–20 amino acids and the molecular weights of the identified proteins were uniformly distributed at different stages. For serum samples, a total of 1,851 types of proteins were identified and 1,580 of them were quantifiable (Supplementary Figure 1A). For LES muscle samples, a total of 6,513 types of proteins were identified and 5,444 of them were quantifiable (Supplementary Figure 1C). The relative standard deviation analysis showed that the quantitative repeatability of serum (Supplementary Figure 1B) and LES muscle samples (Supplementary Figure 1D) met the requirement of further analysis.

### Comparison between achalasia and controls

**Figure 2.**
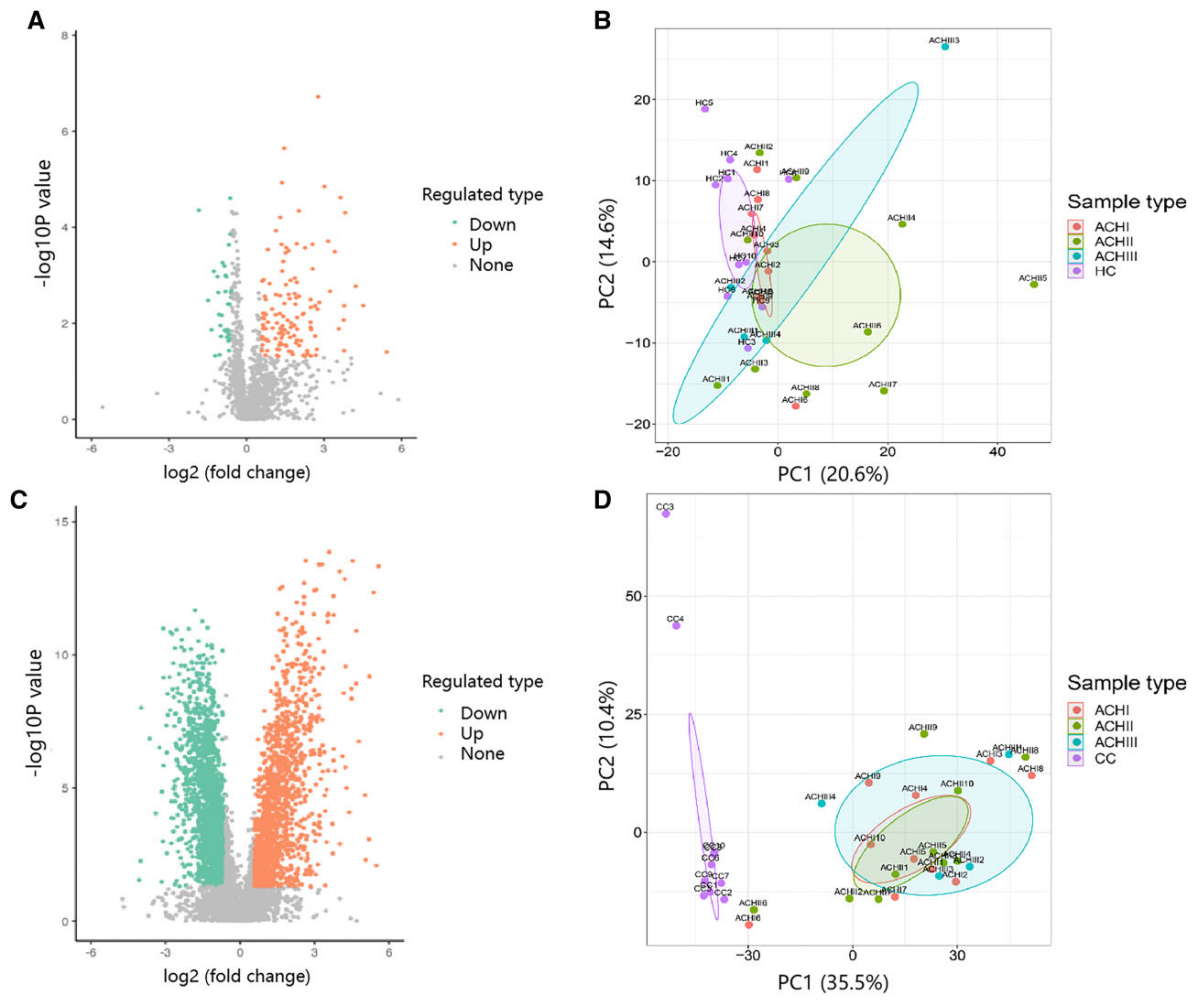
The protein expression pattern. (A) Volcano plot of serum samples from ACH group and HC group. (B) PCA of serum samples. (C) Volcano plot of LES muscle samples from ACH group and CC group. (D) PCA of LES muscle samples. ACH, achalasia; ACH I, type I achalasia; ACH II, type II achalasia; ACH III, type III achalasia; CC, cancer controls; HC, healthy controls; LES, lower esophageal sphincter; PCA, principal component analysis.

**Figure 3.**
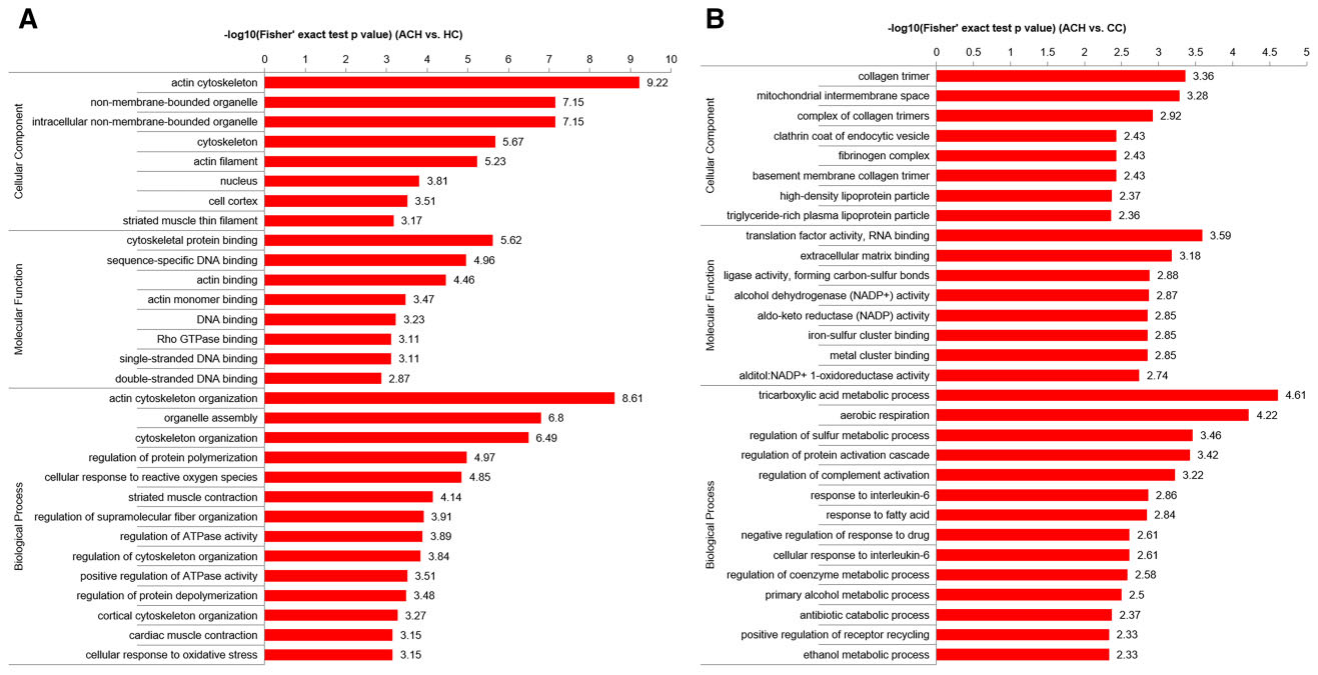
The GO analysis of differentially expressed proteins. (A) Serum samples. (B) LES muscle samples. ACH, achalasia; CC, cancer controls; GO, Gene Ontology; HC, healthy controls; LES, lower esophageal sphincter.

**Figure 4.**
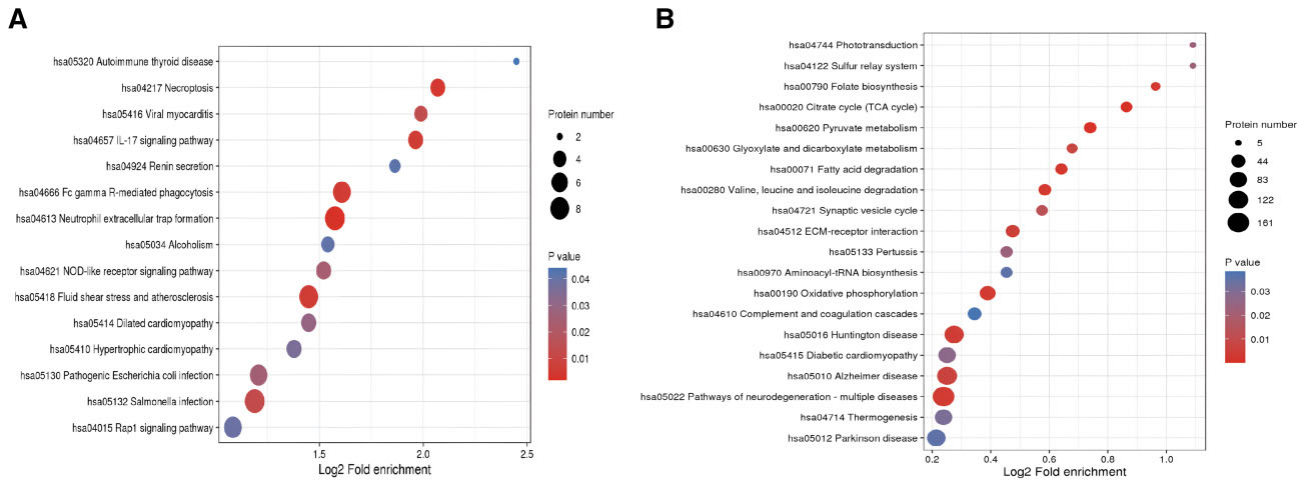
The KEGG pathway enrichment analysis of differentially expressed proteins. (A) Serum samples; (B) LES muscle samples. KEGG, Kyoto Encyclopedia of Genes and Genomes; LES, lower esophageal sphincter.

For serum samples, 128 proteins were upregulated and 36 proteins were downregulated in the achalasia group (Figure 2A). ANOSIM analysis showed that the protein expression pattern of serums from achalasia patients was significantly different from controls (*P *=* *0.020; Figure 2B). The details of differentially expressed proteins of serum are shown in Supplementary Table 1. The GO analysis showed that the differentially expressed serum proteins were mainly involved in the actin cytoskeleton, the cytoskeletal protein binding, and the actin cytoskeleton organization (Figure 3A). The KEGG pathway enrichment analysis showed that the upregulated serum proteins were mainly involved in the neutrophil extracellular trap formation, the necroptosis, the salmonella infection, and the fluid shear stress and atherosclerosis (Figure 4A and Supplementary Figure 2A), whereas the downregulated proteins were mainly involved in the citrate cycle, the fatty acid degradation, the pyruvate metabolism and the valine, and leucine and isoleusine degradation (Figure 4A and Supplementary Figure 2B).

As for muscle samples, 1,314 proteins were upregulated while 1,578 proteins were downregulated in the achalasia group (Figure 2C). LES muscle from achalasia patients showed a marked decrease in nNOS, an inhibitory neurotransmitter synthase (achalasia/controls ratio = 0.013; *P *=* *0.002; Supplementary Table 2). ANOSIM analysis showed that the protein expression pattern of LES muscle from achalasia patients was significantly different from controls (*P *=* *0.001; Figure 2D). The GO analysis showed that the differentially expressed LES muscle proteins were mainly involved in the collagen trimer, the translation factor activity, RNA binding, and the tricarboxylic acid metabolic process (Figure 3B). The KEGG pathway enrichment analysis showed that the upregulated LES muscle proteins were mainly involved in the extracellular matrix (ECM)–receptor interaction, the complement and coagulation cascades, the protein digestion and absorption, and the dilated cardiomyopathy (Figure 4B and Supplementary Figure 2C), whereas the downregulated proteins were mainly involved in the insulin resistance, the vascular smooth muscle contraction, the Epstein–Barr virus infection, and the advanced glycation end-products-receptor for advanced glycation end products (AGE-RAGE) signaling pathway in diabetic complications (Figure 4B and Supplementary Figure 2D).

### Comparison among different achalasia subtypes

To figure out whether different protein expression patterns exist in different subtypes of achalasia, we further conducted a comparison among different achalasia subtypes.

For serum samples, ANOSIM analysis showed that the overall protein expression patterns were significantly different between type I and type II achalasia (*P *=* *0.024; Figure 2B) and between type I and type III achalasia (*P *=* *0.020; Figure 2B). When comparing type II achalasia with type I achalasia, 107 proteins were upregulated (mainly involved in the Fc gamma R-mediated phagocytosis) while 20 proteins were downregulated (mainly involved in the ECM–receptor interaction). When comparing type III achalasia to type I achalasia, 34 proteins were upregulated (mainly involved in the metabolism of xenobiotics by cytochrome) and 33 proteins were downregulated (mainly involved in the transcriptional misregulation in cancer). Although no significant difference of overall proteins was observed between type II and type III achalasia (*P *=* *0.268; Figure 2B), 11 and 23 individual proteins were up- and downregulated separately, and were enriched in neutrophil extracellular trap formation. The details of differentially expressed serum proteins among different subtypes are shown in Supplementary Table 3.

For LES muscle samples, no significant difference was observed in the protein expression patterns among different achalasia subtypes (Figure 2D). When comparing type II achalasia to type I achalasia, 42 proteins were upregulated (mainly involved in the Graft-vs-host disease) while 55 proteins were downregulated (mainly involved in the ubiquitin-mediated proteolysis). When comparing type III achalasia to type I achalasia, 37 proteins were upregulated (mainly involved in the biosynthesis of unsaturated fatty acids) while 49 proteins were downregulated (mainly involved in the ribosome). When comparing type III achalasia to type II achalasia, 62 proteins were upregulated (mainly involved in the biosynthesis of unsaturated fatty acids) while 48 proteins were downregulated (mainly involved in the phosphatidylinositol signaling system). The details of differentially expressed LES muscle proteins among different subtypes are shown in Supplementary Table 4.

**Figure 5.**
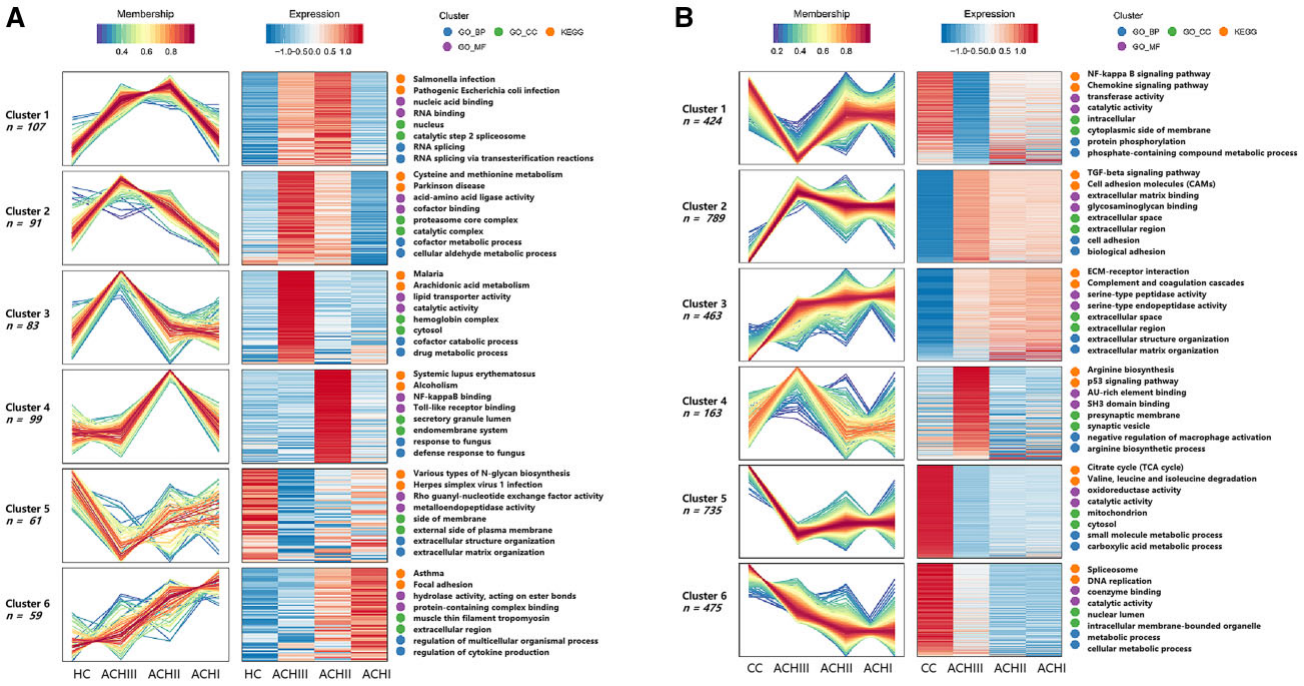
The mfuzz analysis among different groups. (A) Serum samples; (B) LES muscle samples. LES, lower esophageal sphincter; ACH, achalasia; ACH I, type I achalasia; ACH II, type II achalasia; ACH III, type III achalasia; HC, healthy controls; CC, cancer controls.

The mfuzz analysis was used to cluster proteins with similar expression trends among different achalasia subtypes. For serum samples, the protein expression trends are shown in Figure 5A. The proteins in Cluster 6 increased sequentially between the control group, type III, type II, and type I achalasia; these proteins were mainly involved in asthma and focal adhesion (Figure 5A). The protein expression trends of LES muscle samples are shown in Figure 5B. The proteins in Cluster 3 increased sequentially between the control group, type III, type II, and type I achalasia; these proteins were mainly involved in the ECM–receptor interaction and the complement and coagulation cascades (Figure 5B). The proteins in Cluster 6 decreased sequentially between the control group, type III, type II, and type I achalasia; these proteins were mainly involved in the spliceosome and DNA replication (Figure 5B). The specific proteins in the different clusters are shown in Supplementary Tables 5 and 6.

### Proteins changed in both serum and muscle samples

In order to identify potential autoantibodies and explore the consistency of protein profiles between serum and muscle samples, we also analysed which proteins altered in the same directions in both serum and muscle samples. A total of 25 proteins upregulated in both serum and muscle samples of achalasia patients. Most of these proteins were immunity-, infection-, inflammation-, and neurodegeneration-associated. Only one protein, the function of which remained uncertain, was downregulated in both serum and muscle samples of achalasia patients. The details of these proteins are shown in Table 2.

**Table 2.** Proteins change both in serum and lower esophageal sphincter muscle samples

Protein description	Gene name	Ratio (Ach muscle: CC muscle)	Ratio (Ach serum: HC serum)	The KEGG pathways
Thymosin beta-4	*TMSB4X*	3.205	2.841	Regulation of actin cytoskeleton
Tropomodulin-3	*TMOD3*	36.245	4.583	–
Programmed cell death 6-interacting protein	*PDCD6IP*	3.296	3.354	Endocytosis
Peptidyl-prolyl *cis*-*trans* isomerase NIMA-interacting 1	*PIN1*	2.525	1.555	Retinoic acid-inducible gene I-like receptor signaling pathway
High mobility group protein B2	*HMGB2*	3.087	84.356	–
Leukocyte elastase inhibitor	*SERPINB1*	1.587	3.657	–
Transaldolase	*TALDO1*	2.358	2.481	Carbon metabolism; pentose phosphate pathway; biosynthesis of amino acids; metabolic pathways
Histone H1.4	*H1-4*	2.002	297.345	–
SH3 domain-containing kinase-binding protein 1	*SH3KBP1*	2.228	1.631	Endocytosis
Haptoglobin-related protein	*HPR*	3.655	1.716	African trypanosomiasis
Aldehyde dehydrogenase 16 family member A1	*ALDH16A1*	2.714	1.503	–
Azurocidin	*AZU1*	6.357	4.513	Neutrophil extracellular trap formation
Actin-related protein 2/3 complex subunit 2	*ARPC2*	6.545	14.089	Fc gamma R-mediated phagocytosis; tight junction; shigellosis; bacterial invasion of epithelial cells; Salmonella infection; regulation of actin cytoskeleton; pathogenic *Escherichia coli* infection; endocytosis; Yersinia infection
Calmodulin-like protein 3	*CALML3*	2.921	1.674	Ras signaling pathway; vascular smooth muscle contraction; pathways in cancer; apelin signaling pathway; adrenergic signaling in cardiomyocytes; Parkinson disease; pathways of neurodegeneration-multiple diseases; phototransduction; alcoholism; gastric acid secretion; estrogen signaling pathway; glioma; aldosterone synthesis and secretion; tuberculosis; calcium signaling pathway; olfactory transduction; glucagon signaling pathway; neurotrophin signaling pathway; cellular senescence; insulin signaling pathway; lipid and atherosclerosis; dopaminergic synapse; human immunodeficiency virus 1 infection; oxytocin signaling pathway; circadian entrainment; salivary secretion; Alzheimer disease; fluid shear stress and atherosclerosis; phosphatidylinositol signaling system; long-term potentiation; Rap1 signaling pathway; C-type lectin receptor signaling pathway; human cytomegalovirus infection; inflammatory mediator regulation of transient receptor potential channels; gonadotropin releasing hormone signaling pathway; cyclic adenosine monophosphate signaling pathway; melanogenesis; pertussis; renin secretion; Kaposi sarcoma-associated herpes virus infection; amphetamine addiction; cyclic guanosine monophosphate-dependent protein kinase G signaling pathway; oocyte meiosis
Galectin-1	*LGALS1*	2.570	1.557	–
Hematopoietic progenitor cell antigen CD34	*CD34*	5.831	1.604	Cell adhesion molecules; hematopoietic cell lineage
Peptidyl-prolyl *cis*-*trans* isomerase FKBP1A	*FKBP1A*	1.775	2.606	–
LIM and SH3 domain protein 1	*LASP1*	2.894	3.879	–
Integrin alpha-IIb	*ITGA2B*	9.891	1.659	Hypertrophic cardiomyopathy; focal adhesion; neutrophil extracellular trap formation; arrhythmogenic right ventricular cardiomyopathy; pathways in cancer; small cell lung cancer; fluid shear stress and atherosclerosis; regulation of actin cytoskeleton; extracellular matrix–receptor interaction; human papillomavirus infection; phosphatidylinositol 3-kinase/protein kinase B signaling pathway; Ras-associated protein-1; dilated cardiomyopathy; platelet activation; hematopoietic cell lineage
Multimerin-2	*MMRN2*	1.830	1.852	–
Hepatocyte growth factor activator	*HGFAC*	3.031	2.475	–
Macrophage migration inhibitory factor	*MIF*	1.839	2.395	Tyrosine metabolism; phenylalanine metabolism; metabolic pathways
Histone H2A type 2-C	*H2AC20*	2.083	9.384	Systemic lupus erythematosus; neutrophil extracellular trap formation; alcoholism; necroptosis
Membrane cofactor protein	*CD46*	4.243	1.672	Complement and coagulation cascades; measles
Transcobalamin-1	*TCN1*	3.981	1.583	–
Unconventional myosin-XVIIIa	*MYO18A*	0.641	0.641	–

ACH, achalasia; CC, cancer controls; HC, healthy controls; KEGG, Kyoto Encyclopedia of Genes and Genomes.

## Discussion

Achalasia is a primary esophageal motility disorder but the potential molecular pathogenesis remains poorly understood. To the best of our knowledge, this was the first 4D label-free proteomic study in patients with achalasia, with a particular focus on paired samples of both tissue and serum. The data from this study indicated that there were specific protein alterations in both serum and muscle of achalasia, involved in immunity, inflammation, infection, and neurodegeneration pathways. These findings are expected to be able to guide researchers to conduct further mechanism studies and search for potential therapeutic targets.

Only a few studies have currently explored the molecular changes in achalasia. Palmieri *et al.* [25, 26] first identified that gene clusters related to neurological diseases, skeletal and muscular system development, and immunity were over-represented in achalasia patients’ LES muscle in both mRNA and miRNA levels. Seon Kyo Im conducted the first serum proteomic analysis in achalasia patients and found that complements were upregulated in achalasia patients [28]. However, these studies were limited by low-resolution techniques and small sample sizes. None of these studies has examined serum and muscle proteomics simultaneously. Furthermore, no current study has explored the difference in molecular traits among achalasia subtypes. By using a 4D label-free quantification tool, which constitutes dimensions of mass over charge, ion mobility, retention time as well as signal intensity and is able to identify low-abundance protein signals, we have validated previous findings that the molecules involved in achalasia are mainly inflammation-, immunity-, infection-, and neurodegeneration-associated. Moreover, we performed the first systematic screening for potential autoantibodies and the first comparison among achalasia subtypes.

In this study, 164 serum proteins changed significantly in achalasia patients among which the most significantly changed proteins were profilin-1, immunoglobulin heavy variable 3–9, transgelin-2, vasodilator-stimulated phosphoprotein, and galectin-10 (all were upregulated). Also, transgelin-2 has been suggested recently to be a potential biomarker for Alzheimer's disease, which is another neuron degenerative disease [29]. Thus, differentially expressed serum proteins in this study might become the potential biomarker for achalasia (although further validation is still required). Proteomic analysis in LES muscle samples found that the upregulated proteins were mainly involved in the ECM–receptor interaction and the complement and coagulation cascades. Previous studies have found that ECM changes dynamically during nervous system injury. In the acute response phase, the activation of immune cells and extracellular proteases causes degradation of ECM, while in the recovery/chronic phase, the activation of astrocytes and tissue inhibitor of metalloproteinases (TIMP) proteins leads to the upregulated synthesis of ECM molecules [30]. Thus, the upregulation of ECM–receptor interaction in LES of patients with achalasia may represent the chronic stage of neuron injury. In the current study, complement C1q, C1s, C2, C3, C4-A, C4-B, C5, C6, and C9 were all upregulated in the achalasia group. Studies have demonstrated that damaged myelin or autoantibodies can bind and activate C1. Once bound, C1 activates the classical pathway, which injures neurons indirectly by activate inflammation cells and directly through membrane attack complex formation [31, 32]. Furthermore, there has been an explosion of interest in the development of complement-modulating drugs for neurodegenerative diseases [32]. Therefore, further studies on the role of the complement and coagulation cascades pathway in achalasia are essential. This might provide a new therapeutic target for achalasia patients.

Comparisons were also conducted among different achalasia subtypes. Previous histopathology studies have demonstrated that the severity of neurodegeneration differs among different achalasia subtypes [12, 33]. However, there has long been controversy about whether different subtypes of achalasia are caused by different pathogenesis or different processes of the same pathogenesis. In the current study, the ANOSIM analysis showed no significant difference in the overall protein expression patterns in LES muscle among achalasia subtypes, suggesting that there was little difference in LES muscle protein profiles among achalasia subtypes. However, the mfuzz analysis in LES muscle showed that proteins involved in the ECM–receptor interaction increased sequentially between the control group, type III, type II, and type I achalasia. As discussed above, the ECM–receptor interaction pathway changes dynamically during nervous system injury. Thus, for the first time, our study provided molecular evidence for the hypothesis that different subtypes of achalasia are at different stages of the disease.

We also analysed the proteins altered in the same direction in both the muscle and serum samples. This was of great significance for the following studies on the pathogenesis of achalasia. Due to the difficulties in LES muscle specimen acquisition, some studies (including the only proteomic study of achalasia currently) have attempted to reveal the pathogenesis of achalasia by studying the serum of patients. However, we can find out from the current study that the number of quantifiable molecules in serum was significantly lower than that in muscle and there were not many overlap proteins between serum and muscle. Therefore, follow-up studies on the mechanism of achalasia should focus on LES muscle rather than body fluids. In the current study, a total of 25 proteins, most of which were immune-, infection-, inflammation-, and neurodegeneration-associated, were upregulated in both serum and muscle samples of achalasia patients. Although these proteins represented only a minority of the total differentially expressed proteins, these co-elevated proteins may be helpful for searching autoantibodies. Of these 25 proteins, calmodulin-like protein 3 is currently known to be able to bind to neurons and is related to neurodegenerative disease. The release of nitric oxide (NO) by neurons requires nNOS to bind to calmodulin [34] and calmodulin-like protein 3 is a competitive inhibitor of calmodulin. A previous study has demonstrated that although calmodulin-like protein 3 can bind nNOS, nNOS loses the ability to release NO after binding [35]. This might also explain why achalasia only shows loss of inhibitory neurons and no other neurons.

Despite the striking findings, the current study also had some limitations. First, the LES muscles of the normal population were not available in this study. Thus, normal serum controls and normal LES muscle controls were from different populations (normal serum from healthy controls and normal LES from tumor patients). This may lead to some bias in the analysis of proteins that change in both serum and muscle samples. Second, the sample size of type III achalasia was relatively small in this study due to its rarity in clinical practice. This might result in the omission of some potential differentially expressed proteins. Finally, unsupervised clustering was not conducted due to the small sample size in the current study. Further studies are still needed to confirm whether there are new molecular subtypes of achalasia.

In conclusion, by using the high-throughput 4D label-free proteomic measurements, the current study first presented the landscape of tissue and serum proteins in achalasia compared with controls. Distinct protein clusters between types I, II, and III revealed the potential molecular pathways associated with different disease stages. Analysis of proteins changed in the same direction in both muscle and serum samples highlighted the importance of further studies on LES muscle and revealed potential autoantibodies. These findings have provided key molecules related to achalasia pathogenesis that might guide the future development of new therapeutic targets.

## Supplementary Material

goad031_Supplementary_Data
