# Supplementary figures and images for: The molecular pathogenesis of achalasia: a paired lower esophageal sphincter muscle and serum 4D label-free proteomic study
Source: Gastroenterol Rep (Oxf). 2023 Jun 12;11:goad031. doi: 10.1093/gastro/goad031 (PMC10260389; doi:10.1093/gastro/goad031)

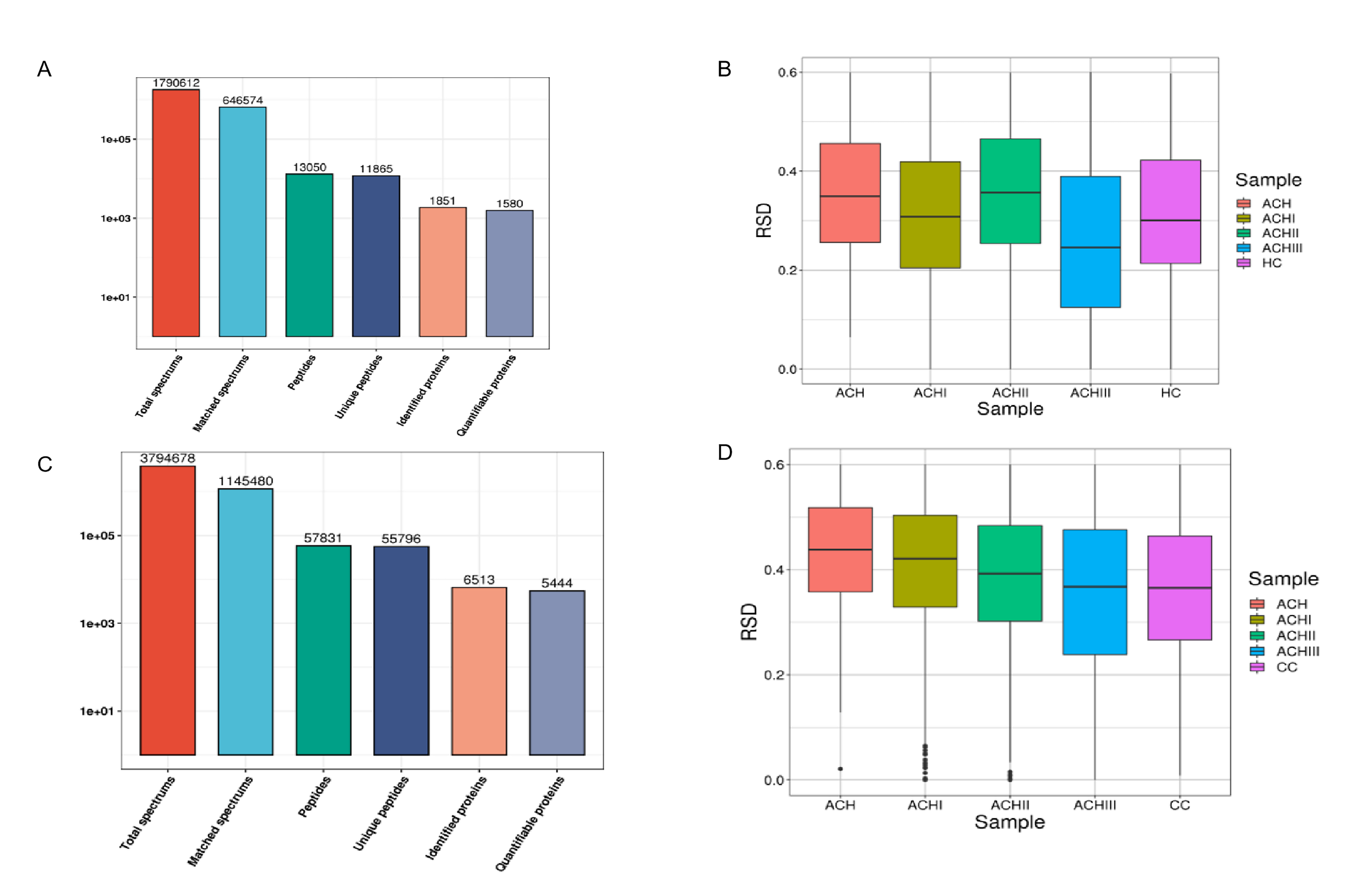

Supplement: goad031_Supplementary_Data [file goad031_supplementary_data.zip › Sup fig 1 final version 20230515.tif]

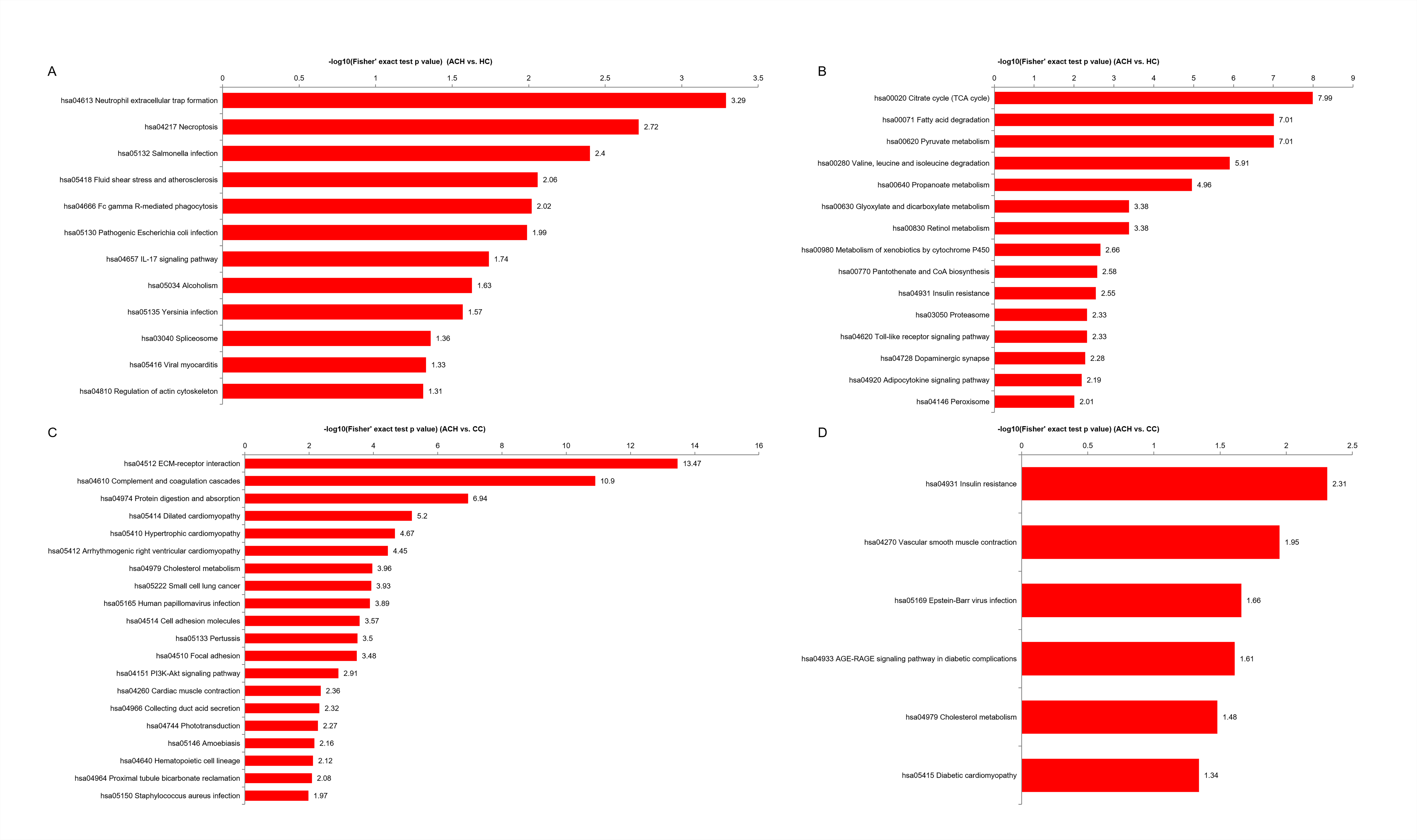

Supplement: goad031_Supplementary_Data [file goad031_supplementary_data.zip › Sup fig 2 final version.tif]
